# *In situ* Vaccination by Direct Dendritic Cell Inoculation: The Coming of Age of an Old Idea?

**DOI:** 10.3389/fimmu.2019.02303

**Published:** 2019-09-25

**Authors:** Luciano Castiello, Eleonora Aricò, Giuseppina D'Agostino, Laura Santodonato, Filippo Belardelli

**Affiliations:** ^1^FaBioCell, Core Facilities, Istituto Superiore di Sanità, Rome, Italy; ^2^Consiglio Nazionale Delle Ricerche, Institute of Translational Pharmacology, Rome, Italy

**Keywords:** dendritic cell (DC), *in situ* vaccination, cancer immunotherapy, checkpoint inhibitor combination therapy, intratumor administration, monocyte derived dendritic cells (MoDC)

## Abstract

For more than 25 years, dendritic cell (DC) based vaccination has flashily held promises to represent a therapeutic approach for cancer treatment. While the vast majority of studies has focused on the use of antigen loaded DC, the intratumoral delivery of unloaded DC aiming at *in situ* vaccination has gained much less attention. Such approach grounds on the ability of inoculated DC to internalize and process antigens directly released by tumor (usually in combination with cell-death-inducing agents) to activate broad patient-specific antitumor T cell response. In this review, we highlight the recent studies in both solid and hematological tumors showing promising clinical results and discuss the main pitfalls and advantages of this approach for endogenous cancer vaccination. Lastly, we discuss how *in situ* vaccination by DC inoculation may fit with current immunotherapy approaches to expand and prolong patient response.

## Introduction

Since the discovery that monocytes, cultured with GM-CSF and IL-4, differentiate into dendritic cells (DC) (1), the idea to use *ex vivo* generated DC to vaccinate cancer patients against tumor antigens has been largely explored (2, 3). Many different protocols have been developed for DC differentiation and/or maturation (4), but there is still a strong need to characterize the relationship between *ex vivo* derived DC and the several *in vivo* circulating DC subsets for which many information are now available in terms of phenotype and functionality (5). Over the last 25 years, hundreds of clinical trials have been performed mostly without showing consistent clinical responses, despite some encouraging results, especially in recent years (6–8). The vast majority of these studies have used mature IL-4-conditioned-DC loaded *ex vivo* with tumor antigens. However, antigen selection has represented one of the major limitations of DC vaccines and it is now widely accepted that broad patient-specific antigen repertoire, using patient tumor lysate or mutanome-derived peptides, represents the most promising DC antigen source (7–10).

An alternative to *ex vivo* antigen loading is represented by the so-called *in situ* vaccination. *In situ* vaccination aims at stimulating DC in the tumor to capture and process antigens released by the tumor and present them to immune cells upon migration to draining lymph node. This approach is receiving renewed interest because of the necessity to expand the antigenic repertoire of T cell responses in the checkpoint blockade therapy era (11–16). Several approaches are being evaluated in early trials, mostly using DC activators directly inoculated within the tumor (13, 16). However, given the low number of pre-existing DC at tumor site, combination therapy with stimulator of hematopoietic differentiation of DC, such as Flt3L, seems to be required for efficient DC activation (17–19).

One way to overcome low intratumoral DC number and ensure a better control of DC phenotype is represented by intratumoral inoculation of *ex vivo* generated DC (*it*DC) aimed at an *in situ* vaccination. First attempts of *it*DC date 20 years back (20–22). Since the initial studies, many promising observations were collected on feasibility and efficacy of *it*DC (23–28), even though they did not get under the spot into the mainstream DC vaccine field. However, recent clinical results (29–31), together with increased interest in *in situ* vaccination to enforce current immunotherapies, highlight *it*DC as a powerful approach that can be rapidly implemented in current checkpoint blockade therapies. In this review, we will present the main results collected in pre-clinical and clinical use of intratumoral delivery of DC and discuss their potential use in combination with current immunotherapy.

## Intratumor Injection of DC: a Platform for Endogenous Vaccination

As professional antigen processing cells, DCs are characterized by the ability to internalize, process and present antigens and potently interact with T cells, thus inducing their activation (32). However, tumors develop several “escape mechanisms” to exclude or reduce immune recognition of tumor-associated antigens, including DC exclusion from tumor microenvironment (33) and inhibition of DC activity (34). Within such an immunosuppressive environment, the injection of *ex vivo* cultured DC represents a valuable approach to overcome some tumor escape mechanisms, process antigens released in necrotic or apoptotic tumor mileu and activate immune response against tumor-associated antigens (35). *it*DC can be potentially applied to almost any tumor type: the only pre-requisite is the possibility to directly inoculate DC in the tumor. In fact, as summarized in Table 1, *it*DC trials have been performed against pancreas (27, 38, 44), liver (27, 37), colorectal (27), prostate (39), esophagus (40), brain (28), skin (26, 42), lung (31, 35), bile duct (27, 35), breast, ovarian, bladder, neuroendocrine (35), renal (43), and hematological tumors (29, 30), and soft tissue sarcoma (41). With the exception of melanoma (which is clearly accessible), the inoculation of DC was guided by ultrasound, computed tomography scan, or endoscopic ultrasound. Only in the setting of a brain tumor was an intraventricular catheter used (28).

**Table 1:** Major clinical trials testing *it*DC.

**DC type**	**Maturation status**	**Clinical setting**	**Tumor pre-conditioning**	**Major findings**	**References**
IL4-DC	Immature	Metastatic melanoma and breast carcinoma	–	Regressing lesions showed lymphocytes infiltration and reactivity against heat shock proteins	(22)
IL-12 transduced IL4-DC	Immature and mature	Advanced metastatic digestive carcinomas	–	IL8 retains DC at tumor site	(27, 36)
IL4-DC	Immature	Refractory hepatoma	Radiotherapy	Systemic antitumor immune response, NK cytotoxicity	(37)
IL4-DC	Immature	Glioma	–	Increased overall survival in patients receiving *it*DC vs. intra dermal DC	(28)
IL4-DC	Immature	Melanoma	±Hyperthermia	Systemic antitumor immune response, enhanced by local hyperthermia	(26)
IL4-DC	Mature	Inoperable pancreatic cancer	Gemcitabine same day	Systemic antitumor immune response and clinical response in combination with lymphokine activated killer cells stimulated with anti-CD3	(38)
IL4-DC	Immature	Prostate	Radiotherapy, hormone therapy	Treatment feasibility, T cells infiltration at tumor site (limited), systemic antitumor immune response (limited)	(39)
IL4-DC	Mature	Esophageal cancer	Chemotherapy	DC are retained at tumor site	(40)
IL4-DC	Immature	Soft tissue sarcoma	Radiotherapy	T cells infiltration at tumor site correlated with antitumor immune response,	(41)
IL4-DC	Immature	Follicular lymphoma	Rituximab and radiotherapy. (GM-CSF given same day)	Systemic antitumor immune response correlated with clinical response	(29)
IFN-DC	Partially mature	Melanoma	Chemotherapy	Systemic antitumor immune response	(42)
Allogeneic IL4-DC	Mature	Metastatic renal cell carcinoma	–	Inflammation at tumor site	(43)
CCL21-transduced IL4-DC	Immature	Non-small cells lung cancer	–	Systemic antitumor immune response, T cells infiltration and increased PD1 expression at tumor site	(31)
GM-CSF DC	Partially mature	Unrespectable, locally advanced, or metastatic solid tumors	–	Increased production of specific cytokines by DC correlated with clinical efficacy	(35)
IFN-DC	Partially mature	Follicular lymphoma	Rituximab	Systemic antitumor immune response; abscopal effect	(30)

**Figure 1:**
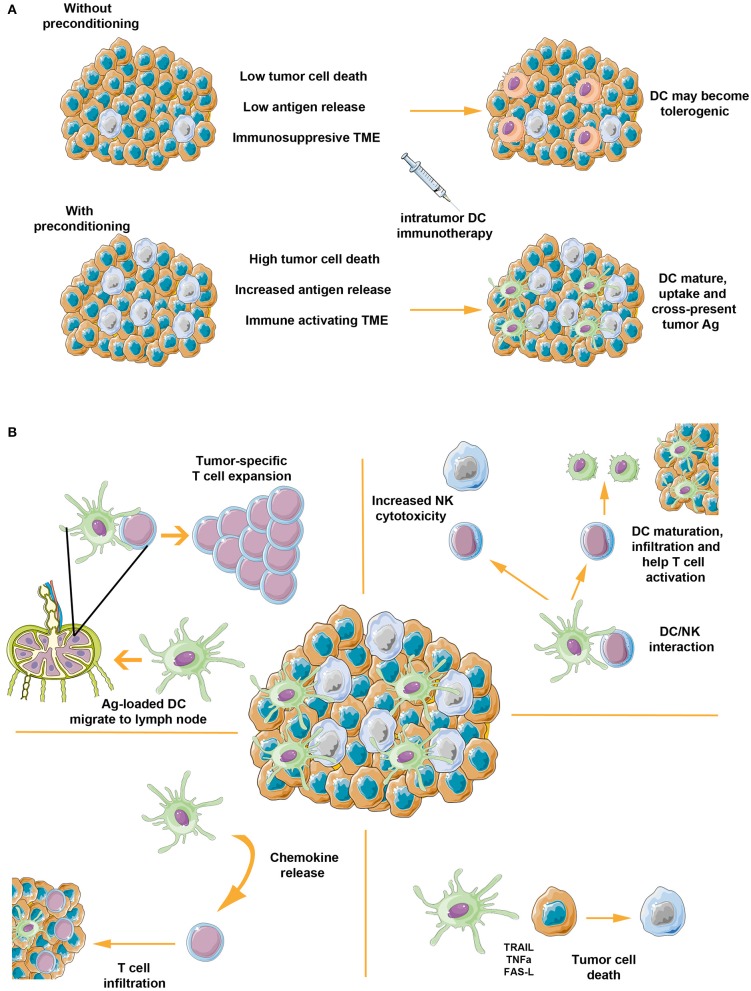
Intratumor inoculation of DC: the importance of preparing tumor microenvironment and its multiple ways of action. **(A)** In the absence of any treatment, tumors are characterized by low levels of basal apoptotic/necrotic cells and an immunosuppressive microenvironment. Within this setting, *it*DC might become tolerogenic, thus increasing tumor immunosuppressive features and eventually causing a detrimental effect. Tumor preconditioning with immunogenic cell death agents, instead, can enhance tumor cells apoptosis, resulting in increased release of tumor-associated antigens and immune activating signals. In this scenario, intratumor inoculated DC sense proinflammatory and immune activating signals, process tumor antigens, and activate antitumor response. **(B)** *it*DC can activate immune response by acting on several mechanisms. After loading tumor released antigens, mature DC migrate to draining lymph node where they interact with T cells and lead to increased clonality and richness of antitumor T cell responses. The DC interaction with intratumor NK cells can activate their cytotoxic activity, which in turn can activate a positive feedback on DC themselves by boosting their maturation, their infiltration and favoring DC/CD4^+^ T cell interactions. *it*DC can also increase infiltration of T cells by secreting chemokines and exert direct cytotoxic effect, resulting in increased tumor cell death and, more importantly, increased release of tumor antigens. The figure was made using the Servier Medical ART set by Servier.

Even though basal tumor apoptosis/necrosis can be exploited (27, 35, 44), *it*DC vaccination strongly benefits from tumor pre-treatment with death-inducing agents, because of the increased release of tumor antigens (Figure 1A) (24, 26). Among the pre-conditioning regimens used, the ones causing immunogenic cell death are clearly preferred, because they couple the release of tumor antigens with DC activating signals (45–47). However, as shown by Teitz-Tennenbaum, radiotherapy (RT), inducing calreticulin exposure and other activating signals (48, 49), stimulates DC processing ability, homing to lymph node, and their ability to stimulate T cells even when RT was not inducing tumor cell death (50). This point indicates that *it*DC can strongly benefit, not only from tumor pre-conditioning with immunogenic cell death treatments, but also with regimens that simply increase immunogenicity of tumors, thus enlarging the range of possible agents that can be used.

Among the several pre-conditioning regimens, RT represents the broadest applicable one considering also the ease of adding *it*DC into already well-established RT regimens (24, 37, 41). However, clinical trials have also been performed using local hyperthermia (26), systemic chemotherapy (40), and tumor-targeting monoclonal antibodies (29, 30) (Table 1). While an ideal pre-condition approach should be tailored to tumor type, the use of tumor-targeting monoclonal antibodies raises some fascinating advantages and synergies. First, considering that DC are endowed with antibody-dependent cell-mediated cytotoxicity (51), the two treatments could directly synergize. In fact, we have shown that direct cytotoxic activity of DC against the lymphoma cell line Karpas-422 was increased after rituximab pre-treatment (30). Second, it has been shown that, for a successful monoclonal therapy, an NK-DC crosstalk needs to be mounted, where NK cell activation leads to increased cross-presentation and maturation of DC, thus resulting in antitumor T cell activation (52, 53). Therefore, *it*DC might boost such crosstalk, leading to increased NK cell activity and stronger adaptive antitumor immune responses. Third, combining monoclonal antibody with *it*DC can potentially lead to *in situ* vaccination targeted against clinically relevant, rare cells within the tumor, such as cancer stem cells. In fact, even though monoclonal antibodies recognizing cancer stem cells have not yet shown promising results (54), the possibility to directly target CSC with monoclonal antibodies, in combination with *it*DC to activate T cell immunity against CSC, may hold great promises and deserves future testing.

### *it*DC, a 360-Degree Immunotherapy

Even though *it*DC based therapy is principally aimed at direct *in situ* vaccination, several complementary immunotherapy effects can also result (Figure 1B). As clearly shown by pre-clinical studies on *it*DC, NK cells can be directly targeted and activated by *it*DC (55, 56). In fact, depletion of NK cells led to impaired efficacy of *it*DC. This is not surprising in light of the tight crosstalk existing between DC and NK cells (57, 58). On one side, DC can potently activate NK cell cytotoxicity against tumor cells through secreted cytokines and cell-to-cell contact (59, 60). This, in turn, stimulates NK cells to secrete CCL5, XCL1, and Flt3L in the tumor (61, 62), thus promoting natural DC infiltration and additional cross-priming of tumor-associated antigen (63). On the other side, NK cells can strongly enhance DC maturation and IL-12 production, stimulate CD4 T cell response and, through IFN-γ, help DC-driven Th1 polarization (64, 65). In line with this crosstalk, it has been observed that high levels of NK cells after DC vaccination correlated with clinical response in acute myeloid leukemia (66) and advanced hepatoma patients (37).

Another complementary effect of *it*DC that should be taken into account is the ability of DC (especially upon maturation) to secrete several chemokines that can favor the infiltration of T cells and endogenous DC in the tumor microenvironment. This possibility has been recently tested in renal cell carcinoma by injecting allogeneic DC, therefore, excluding any direct vaccination effect but rather potentiating inflammatory-related signals due to cell allogeneity (43). Notably, the authors observed a high level of T cell infiltration and induction of tumor specific T cell responses in three out of 11 evaluable patients. Even more interestingly, despite clinical responses not being registered, an unexpected response consisting in high infiltration of T cells was observed in patients subsequently treated with tyrosine kinase inhibitors, thus suggesting a synergistic effect of the allo-*it*DC with tyrosine kinase inhibitors, possibly mediated by their effect against Treg and MDSC (43, 67). This approach has been additionally tested in advanced hepatocellular carcinoma patients where induction of tumor-specific immune activation in a substantial number of patients was observed (68). Alternative to the use of allogeneic cells, another approach to boost *it*DC ability to inflame the tumor and/or stimulate immune cells has been tested by genetically modifying DC for constitutive expression of activating factors. DC transduced for the expression of IL-7, IL-12, IL-15, IFN-α, and CCL21 have all been tested in pre-clinical models of *it*DC showing encouraging results (20, 21, 25, 55, 69, 70), even though clinical experience with IL-12 transduced DC showed limited success (27, 36).

Lastly, despite being usually neglected, DC are also characterized by direct tumoricidal activity, which, in the context of *it*DC, might result in additional tumor cell death and more importantly in increased release of tumor antigens and damage associated molecular patterns, thus potentiating immune reactivation. In fact, *ex vivo* generated DC, circulating conventional DC and plasmacytoid DC exert direct cytotoxicity against tumor cells (51). This ability has been demonstrated against a large variety of cancer cell lines and can be mediated by both cell-to-cell signals and secreted factors. While TRAIL is the major signal by which DC exert their tumoricidal activity (71), TNFα, FAS-L, caspase-8, IFN-γ, and Granzyme B can also play a role (51).

### The Ideal DC Phenotype for *it*DC Immunotherapy: Lessons Learned

Conversely to the classical antigen-loaded DC vaccination approach, for which many different protocols for DC differentiation and maturation have been developed and compared (4, 72, 73), minimal discussion has been raised regarding the phenotype of DC to be used for intratumoral inoculation. Initial studies focused on the use of immature DC to take advantage of enhanced phagocytic and antigen processing ability of these cells over the mature counterparts (21, 25, 74). However, in absence of strong DC activating stimuli (i.e., when tumor pre-conditioning is not performed or not inducing strong immunogenic cell death), immature DC can have a detrimental effect exerting more immunosuppressive rather than immunostimulatory activity (75–78). Therefore, a semi-mature phenotype may be preferable to couple phagocytic activity with the predefined immunostimulatory mature phenotype (35). However, additional immunotherapy effects of *it*DC (see above) should also be taken into account.

While other protocols to generate semi-mature DC have been developed (79, 80), we opted for DC differentiated in the presence of IFN-α instead of IL-4 (30, 42). These cells (named as IFN-DC) have been discovered by our group almost 20 years ago and are characterized by a partially mature phenotype and are endowed with a high migratory behavior and immunostimulatory ability (81–83). They have been shown to be more efficient than conventional IL-4-DC in internalizing tumor antigens and in the cross-priming of CD8^+^ T cells, thus promoting anti-tumor immune responses (84, 85). Moreover, it has been shown that IFN-DC can promote efficient NK cell activation, increase expression of cytotoxicity receptors, and stimulate extensive IFN-γ production by NK cells (86). Interestingly, in two different clinical trials, we observed induction of long-term T cell immune response against tumor associated antigens upon *it*DC immunotherapy using IFN-DC (30, 42).

### The Coming of Age of *it*DC: Clinical and Immunological Responses in Recent Trials

While initial attempts of *it*DC showed limited success, recent trials have convincingly shown not only safety and feasibility of *it*DC immunotherapy, but also clear-cut clinical and immunological responses in a high percentage of patients (Table 1). In two studies in follicular lymphoma patients, *it*DC in combination with low-dose rituximab alone (30) or in combination with low-dose rituximab, plus local radiotherapy, and GM-CSF (29), showed induction of 50 and 36%, respectively, of objective clinical responses in treated and untreated lesions. Notably, in both studies', induction of both CD8 and CD4 antitumor specific responses were collected and the magnitude of immune activation appeared to correlate with clinical response. Despite several differences between the two trials (type of DC used, pre-conditioning regimen, treatment schedule), these two studies clearly indicate that follicular lymphoma is particularly suited for *it*DC and that this immunotherapy approach is worth being tested in phase II-III clinical trials.

In another interesting clinical study, Lee et al. used CCL21 transduced DC in NSCLC (31). Despite minimal clinical effects being recorded, induction of T cell responses against tumor associated antigens were observed in 6/16 patients and, in four patients, induction of humoral response was reported. However, more noteworthy is the observation that, with only two DC inoculations, an increase in CD8 T cell infiltration was observed in 56% of patients and that this was correlated with increased expression of checkpoint inhibitors (31). Similar results were collected by another study using activated DC in several tumor types, showing that increased PD-L1 expression in the majority of patients usually paired by T cell infiltration (35). Thus, altogether both studies suggest that *it*DC itself increased PD-L1 expression as a result of antigen recognition and CD8 T cell infiltration at the tumor site, clearly pointing to synergies that can result by combining *it*DC and checkpoint blockade.

### *it*DC for Checkpoint Blockade Immunotherapy: Arming T Cells While Preparing the Battlefield

**Figure 2:**
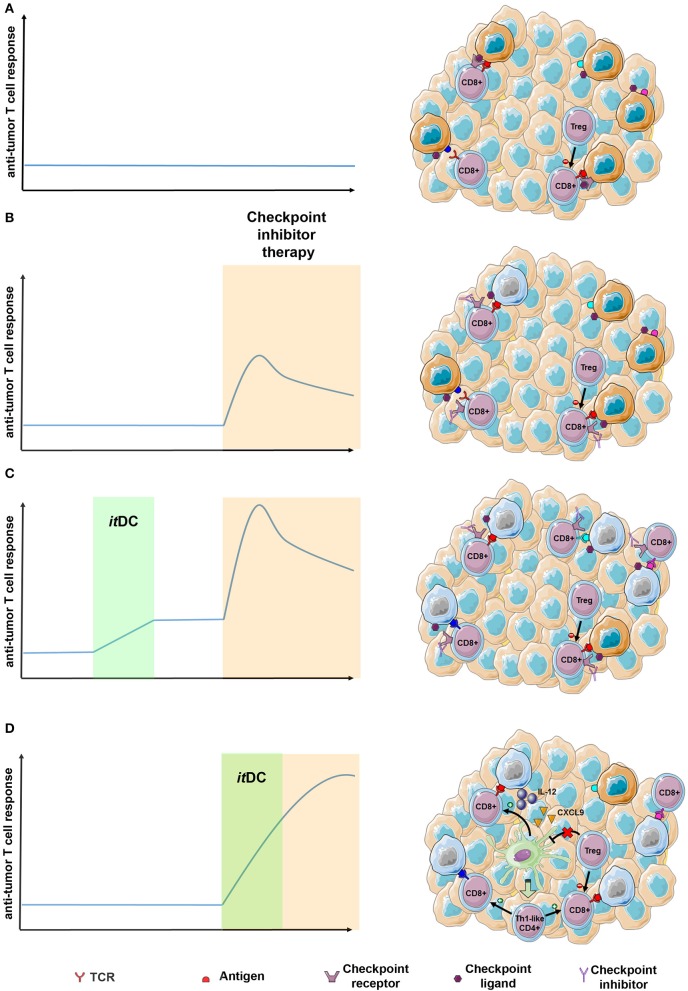
Expected advantages of integrating the “*it*DC” approach in the context of the checkpoint blockade therapy. **(A)** In poorly immunogenic tumors, T cell response is usually low due to multiple checkpoint inhibitors expressed within the tumor microenvironment and immunosuppressive cells. **(B)** Checkpoint blockade therapy may result in increased antitumor T cell response of pre-existing antitumor clones. However, reduced richness and clonality, together with the presence of Treg cell limits checkpoint blockade efficacy, thus resulting in short-lived responses in the majority of patients. **(C)** *it*DC immunotherapy administered prior to checkpoint blockade therapy may lead to increased T cell clonality and richness. In this setting, subsequent checkpoint inhibitor administration is expected to lead to broader antitumor T cell activation. **(D)** *it*DC immunotherapy taking place during checkpoint blockade therapy may boost intratumor T cell activation by secreting IL-12, CXCL9. Additionally, it could overcome inhibitory signals from Treg cells, thus unleashing activation and infiltration of Th1-like CD4^+^ T cells that can further potentiate antitumor T cell response (97–99). The figure was made using the Servier Medical ART set by Servier.

Checkpoint blockade is revolutionizing cancer therapy with impressive long-term responses in a large variety of tumors. However, the majority of patients still do not benefit from this therapy because of either primary or secondary resistance (87). Several factors have been identified playing a role behind response to checkpoint blockade: tumor mutation burden (88), PDL1 expression (89, 90), T cell inflamed microenvironment (91), T cell repertoire richness and clonality (92), HLA-I diversity (93), intestinal microbiota (94, 95), and specific mutations have all been identified as potential markers with prognostic or predictive value in checkpoint blockade therapy (87). Additionally, cross-priming and CXCL9/10 secretion mediated by intratumoral CD103^+^ BATF3-dependent dendritic cells has also been correlated with response to checkpoint blockade (17, 96). Notably, *it*DC studies have already been shown to lead to increased tumor PDL1 expression and increased T cell responses in several tumor types (31, 35). In our recent study, combining NGS technology with *in silico* prediction, we analyzed T cell responses against patient specific mutations in follicular lymphoma patients before and after *it*DC and observed an increase in pre-existing T cell responses in some patients. This, thus, indicates increased T cell clonality and induction (within the limit of assay detection) of *de novo* T cell response, suggesting increased T cell richness of antigenic repertoire (30). Altogether, the evidences gained in clinical studies and animal models with *it*DC imply that checkpoint blockade therapy could be enhanced by prior itDC immunotherapy (Figure 2).

Interestingly, the role of intratumoral DC subsets in response to checkpoint blockade therapy has recently emerged, depicting two independent axis: an NK/cDC1/IL-12-CXCL9 axis needed for effective CD8 T cell response and a Treg/cDC2 axis for effective CD4 T cell response. In one study mainly focused on melanoma, Barry et al. have described the role of intratumoral NK cells in increasing cDC1 abundance within a tumor microenvironment by secreting FLT3LG, showing that the abundance of both populations positively correlates with the response to checkpoint blockade therapy (62). Further, recent literature has unraveled how intratumoral cDC1 “license” CD8 response during checkpoint blockade by secreting IL-12 and CXCL9, potentiating T cell activation (97, 98). On the other side, Binnewies et al. have discovered that levels of cDC2 populations relative to Treg abundance within tumor microenvironment are responsible of infiltration by CD4 T cells and correlate with the response to checkpoint blockade therapy (99). Whether *it*DC during checkpoint blockade therapy could potentiate T cell activity by secreting IL-12, CXCL9 or by overcoming inhibitory activity of Treg has not yet been analyzed. However, it is reasonable to expect that activated *it*DC will sum up with intratumoral DC in sustaining T cell responses during checkpoint blockade therapy (Figure 2).

## Conclusions and Perspectives

The intratumoral delivery of DC has been tested in several different clinical settings, where it has been proved to not only be feasible and safe, but also to be capable of enhancing and/or inducing a tumor-specific immune response.

The possibility to exploit, by an endogenous vaccination strategy, the broad tumor antigen repertoire promptly released by immunogenic tumor pre-conditioning, makes *it*DC a versatile cell therapy, potentially overcoming some of the limitations of therapies based on *ex-vivo* antigen loaded DC, such as the lack of dominant tumor antigens, the availability of tumor samples, and the possible emergence of neo-antigens.

Although the limited number of patients enrolled in phase I studies demands a prudent evaluation of the observed clinical results, data collected so far look promising, and encourages the application of *it*DC to hitherto unexplored clinical settings. More research efforts should yet be devoted to the identification of the optimal DC types to be used in *it*DC strategies, as well as of the most effective strategies for tumor microenvironment pre-conditioning tailored for specific clinical settings. Of note, the accumulating knowledge on their mechanism of action, by showing that *it*DC can affect tumor microenvironment at different levels (including cytokine release and NK cells stimulation), also provides the rationale for their use in combination with immunotherapy approaches currently used in oncology, such as immune checkpoint inhibitors. Based on the evidence available, summarized in this review, we envisage that *it*DC, administered prior or in concomitance with checkpoint inhibitors, by triggering a broader and more effective antitumor immune response, can not only prolong their efficacy, but also provide clinical benefit to patients showing limited responsiveness to checkpoint inhibitors *per se*.

## Author Contributions

LC, EA, and FB wrote the manuscript. LC and EA prepared the figures. All authors contributed to manuscript revision, read, and approved the submitted version.

### Conflict of Interest

The authors declare that the research was conducted in the absence of any commercial or financial relationships that could be construed as a potential conflict of interest. The handling editor declared a past co-authorship with one of the authors FB.

## References

[1] Sallusto F Lanzavecchia A. Efficient presentation of soluble antigen by cultured human dendritic cells is maintained by granulocyte/macrophage colony-stimulating factor plus interleukin 4 and downregulated by tumor necrosis factor alpha. J Exp Med. (1994) 179:1109–18. 10.1084/jem.179.4.1109 8145033 PMC2191432

[2] Garg AD Vara Perez M Schaaf M Agostinis P Zitvogel L Kroemer G. Trial watch: dendritic cell-based anticancer immunotherapy. Oncoimmunology. (2017) 6:e1328341. 10.1080/2162402X.2017.1328341 28811970 PMC5543823

[3] Butterfield LH. Dendritic cells in cancer immunotherapy clinical trials: are we making progress? Front Immunol. (2013) 4:454. 10.3389/fimmu.2013.00454 24379816 PMC3861778

[4] Castiello L Sabatino M Jin P Clayberger C Marincola FM Krensky AM. Monocyte-derived DC maturation strategies and related pathways: a transcriptional view. Cancer Immunol Immunother. (2011) 60:457–66. 10.1007/s00262-010-0954-6 21258790 PMC3086891

[5] Villani A-C Satija R Reynolds G Sarkizova S Shekhar K Fletcher J. Single-cell RNA-seq reveals new types of human blood dendritic cells, monocytes, and progenitors. Science. (2017) 356:eaah4573. 10.1126/science.aah4573 28428369 PMC5775029

[6] Anguille S Smits EL Lion E van Tendeloo VF Berneman ZN. Clinical use of dendritic cells for cancer therapy. Lancet Oncol. (2014) 15:e257–67. 10.1016/S1470-2045(13)70585-0 24872109

[7] Liau LM Ashkan K Tran DD Campian JL Trusheim JE Cobbs CS First results on survival from a large Phase 3 clinical trial of an autologous dendritic cell vaccine in newly diagnosed glioblastoma. J Transl Med. (2018) 16:142 10.1186/s12967-018-1507-6 29843811 PMC5975654

[8] Tanyi JL Bobisse S Ophir E Tuyaerts S Roberti A Genolet R. Personalized cancer vaccine effectively mobilizes antitumor T cell immunity in ovarian cancer. Sci Transl Med. (2018) 10:eaao5931. 10.1126/scitranslmed.aao5931 29643231

[9] Carreno BM Magrini V Becker-Hapak M Kaabinejadian S Hundal J Petti AA. A dendritic cell vaccine increases the breadth and diversity of melanoma neoantigen-specific T cells. Science. (2015) 348:803–8. 10.1126/science.aaa3828 25837513 PMC4549796

[10] Garg AD Coulie PG Van den Eynde BJ Agostinis P. Integrating next-generation dendritic cell vaccines into the current cancer immunotherapy landscape. Trends Immunol. (2017) 38:577–93. 10.1016/j.it.2017.05.006 28610825

[11] Hammerich L Binder A Brody JD. *In situ* vaccination: cancer immunotherapy both personalized *and* off-the-shelf. Mol Oncol. (2015) 9:1966–81. 10.1016/j.molonc.2015.10.016 26632446 PMC5528727

[12] Chiang CL-L Kandalaft LE. *In vivo* cancer vaccination: which dendritic cells to target and how? Cancer Treat Rev. (2018) 71:88–101. 10.1016/j.ctrv.2018.10.012 30390423 PMC6295330

[13] Aznar MA Tinari N Rullán AJ Sánchez-Paulete AR Rodriguez-Ruiz ME Melero I. Intratumoral delivery of immunotherapy—act locally, think globally. J Immunol. (2017) 198:31–9. 10.4049/jimmunol.1601145 27994166

[14] Brody JD Goldstein MJ Czerwinski DK Levy R. Immunotransplantation preferentially expands T-effector cells over T-regulatory cells and cures large lymphoma tumors. Blood. (2009) 113:85–94. 10.1182/blood-2008-05-155457 18812472 PMC2614645

[15] Kim YH Gratzinger D Harrison C Brody JD Czerwinski DK Ai WZ. *In situ* vaccination against mycosis fungoides by intratumoral injection of a TLR9 agonist combined with radiation: a phase 1/2 study. Blood. (2012) 119:355–63. 10.1182/blood-2011-05-355222 22045986 PMC3257006

[16] Frank MJ Reagan PM Bartlett NL Gordon LI Friedberg JW Czerwinski DK. *In situ* vaccination with a TLR9 agonist and local low-dose radiation induces systemic responses in untreated indolent lymphoma. Cancer Discov. (2018) 8:1258–69. 10.1158/2159-8290.CD-18-0743 30154192 PMC6171524

[17] Salmon H Idoyaga J Rahman A Leboeuf M Remark R Jordan S. Expansion and activation of CD103 + dendritic cell progenitors at the tumor site enhances tumor responses to therapeutic PD-L1 and BRAF inhibition. Immunity. (2016) 44:924–38. 10.1016/j.immuni.2016.03.012 27096321 PMC4980762

[18] Sánchez-Paulete AR Teijeira Á Quetglas JI Rodríguez-Ruiz ME Sánchez-Arráez Á Labiano S. Intratumoral immunotherapy with XCL1 and sFlt3L encoded in recombinant semliki forest virus–derived vectors fosters dendritic cell–mediated T-cell cross-priming. Cancer Res. (2018) 78:6643–54. 10.1158/0008-5472.CAN-18-0933 30297531

[19] Hammerich L Marron TU Upadhyay R Svensson-Arvelund J Dhainaut M Hussein S. Systemic clinical tumor regressions and potentiation of PD1 blockade with *in situ* vaccination. Nat Med. (2019) 25:814–24. 10.1038/s41591-019-0410-x 30962585

[20] Sharma S Miller P Stolina M Zhu L Huang M Paul R. Multicomponent gene therapy vaccines for lung cancer: effective eradication of established murine tumors *in vivo* with interleukin-7/herpes simplex thymidine kinase-transduced autologous tumor and *ex vivo* activated dendritic cells. Gene Ther. (1997) 4:1361–70. 10.1038/sj.gt.3300531 9472560

[21] Melero I Duarte M Ruiz J Sangro B Galofré JC Mazzolini G. Intratumoral injection of bone-marrow derived dendritic cells engineered to produce interleukin-12 induces complete regression of established murine transplantable colon adenocarcinomas. Gene Ther. (1999) 6:1779–84. 10.1038/sj.gt.3301010 10516729

[22] Triozzi PL Khurram R Aldrich WA Walker MJ Kim JA Jaynes S. Intratumoral injection of dendritic cells derived *in vitro* in patients with metastatic cancer. Cancer. (2000) 89:2646–54. 10.1002/1097-0142(20001215)89:12<2646::AID-CNCR18>3.0.CO;2-A 11135227

[23] Yu B Kusmartsev S Cheng F Paolini M Nefedova Y Sotomayor E. Effective combination of chemotherapy and dendritic cell administration for the treatment of advanced-stage experimental breast cancer. Clin Cancer Res. (2003) 9:285–94. 12538481

[24] Teitz-Tennenbaum S Li Q Rynkiewicz S Ito F Davis MA McGinn CJ. Radiotherapy potentiates the therapeutic efficacy of intratumoral dendritic cell administration. Cancer Res. (2003) 63:8466–75. 14679011

[25] Miller PW Sharma S Stolina M Butterfield LH Luo J Lin Y. Intratumoral administration of adenoviral interleukin 7 gene-modified dendritic cells augments specific antitumor immunity and achieves tumor eradication. Hum Gene Ther. (2000) 11:53–65. 10.1089/10430340050016157 10646639

[26] Guo J Zhu J Sheng X Wang X Qu L Han Y. Intratumoral injection of dendritic cells in combination with local hyperthermia induces systemic antitumor effect in patients with advanced melanoma. Int J Cancer. (2007) 120:2418–25. 10.1002/ijc.22551 17294445

[27] Mazzolini G Alfaro C Sangro B Feijoó E Ruiz J Benito A. Intratumoral injection of dendritic cells engineered to secrete interleukin-12 by recombinant adenovirus in patients with metastatic gastrointestinal carcinomas. J Clin Oncol. (2005) 23:999–1010. 10.1200/JCO.2005.00.463 15598979

[28] Yamanaka R Homma J Yajima N Tsuchiya N Sano M Kobayashi T. Clinical evaluation of dendritic cell vaccination for patients with recurrent glioma: results of a clinical phase I/II trial. Clin Cancer Res. (2005) 11:4160–7. 10.1158/1078-0432.CCR-05-0120 15930352

[29] Kolstad A Kumari S Walczak M Madsbu U Hagtvedt T Bogsrud TV Sequential intranodal immunotherapy induces anti-tumor immunity and correlated regression of disseminated follicular lymphoma. Blood. (2015) 125:82–9. 10.1182/blood-2014-07-592162 25293773

[30] Cox MC Castiello L Mattei M Santodonato L D'Agostino G Muraro E. Clinical and antitumor immune responses in relapsed/refractory follicular lymphoma patients after intranodal injections of IFNα-dendritic cells and rituximab. Clin Cancer Res. (2019) 25:5231–41. 10.1158/1078-0432.CCR-19-0709 31171545

[31] Lee JM Lee M-H Garon E Goldman JW Salehi-Rad R Baratelli FE. Phase I trial of intratumoral injection of *CCL21* gene–modified dendritic cells in lung cancer elicits tumor-specific immune responses and CD8 ^+^ T-cell infiltration. Clin Cancer Res. (2017) 23:4556–68. 10.1158/1078-0432.CCR-16-2821 28468947 PMC5599263

[32] Steinman RM. Dendritic cells: understanding immunogenicity. Eur J Immunol. (2007) 37:S53–60. 10.1002/eji.200737400 17972346

[33] Binnewies M Roberts EW Kersten K Chan V Fearon DF Merad M. Understanding the tumor immune microenvironment (TIME) for effective therapy. Nat Med. (2018) 24:541–50. 10.1038/s41591-018-0014-x 29686425 PMC5998822

[34] Böttcher JP Reis E Sousa C. The role of type 1 conventional dendritic cells in cancer immunity. Trends Cancer. (2018) 4:784–92. 10.1016/j.trecan.2018.09.001 30352680 PMC6207145

[35] Subbiah V Murthy R Hong DS Prins RM Hosing C Hendricks K. Cytokines produced by dendritic cells administered intratumorally correlate with clinical outcome in patients with diverse cancers. Clin Cancer Res. (2018) 24:3845–56. 10.1158/1078-0432.CCR-17-2707 30018119 PMC6449174

[36] Feijoó E Alfaro C Mazzolini G Serra P Peñuelas I Arina A. Dendritic cells delivered inside human carcinomas are sequestered by interleukin-8. Int J Cancer. (2005) 116:275–81. 10.1002/ijc.21046 15800914

[37] Chi K-H Liu S-J Li C-P Kuo H-P Wang Y-S Chao Y. Combination of conformal radiotherapy and intratumoral injection of adoptive dendritic cell immunotherapy in refractory hepatoma. J Immunother. (2005) 28:129–35. 10.1097/01.cji.0000154248.74383.5e 15725956

[38] Hirooka Y Itoh A Kawashima H Hara K Nonogaki K Kasugai T. A combination therapy of gemcitabine with immunotherapy for patients with inoperable locally advanced pancreatic cancer. Pancreas. (2009) 38:e69–74. 10.2958/suizo.24.632 19276867

[39] Finkelstein SE Rodriguez F Dunn M Farmello M-J Smilee R Janssen W. Serial assessment of lymphocytes and apoptosis in the prostate during coordinated intraprostatic dendritic cell injection and radiotherapy. Immunotherapy. (2012) 4:373–82. 10.2217/imt.12.24 22512631 PMC4241355

[40] Fujiwara S Wada H Miyata H Kawada J Kawabata R Nishikawa H. Clinical trial of the intratumoral administration of labeled DC combined with systemic chemotherapy for esophageal cancer. J Immunother. (2012) 35:513–21. 10.1097/CJI.0b013e3182619cb4 22735809

[41] Finkelstein SE Iclozan C Bui MM Cotter MJ Ramakrishnan R Ahmed J. Combination of external beam radiotherapy (EBRT) with intratumoral injection of dendritic cells as neo-adjuvant treatment of high-risk soft tissue sarcoma patients. Int J Radiat Oncol Biol Phys. (2012) 82:924–32. 10.1016/j.ijrobp.2010.12.068 21398051 PMC4241354

[42] Rozera C Cappellini GA D'Agostino G Santodonato L Castiello L Urbani F. Intratumoral injection of IFN-alpha dendritic cells after dacarbazine activates anti-tumor immunity: results from a phase I trial in advanced melanoma. J Transl Med. (2015) 13:139. 10.1186/s12967-015-0473-5 25933939 PMC4438625

[43] Laurell A Lönnemark M Brekkan E Magnusson A Tolf A Wallgren AC. Intratumorally injected pro-inflammatory allogeneic dendritic cells as immune enhancers: a first-in-human study in unfavourable risk patients with metastatic renal cell carcinoma. J Immunother Cancer. (2017) 5:52. 10.1186/s40425-017-0255-0 28642820 PMC5477104

[44] Endo H Saito T Kenjo A Hoshino M Terashima M Sato T. Phase I trial of preoperative intratumoral injection of immature dendritic cells and OK-432 for resectable pancreatic cancer patients. J Hepatobiliary Pancreat Sci. (2012) 19:465–75. 10.1007/s00534-011-0457-7 21983893

[45] Galluzzi L Buqué A Kepp O Zitvogel L Kroemer G. Immunogenic cell death in cancer and infectious disease. Nat Rev Immunol. (2017) 17:97–111. 10.1038/nri.2016.107 27748397

[46] Suek N Campesato LF Merghoub T Khalil DN. Targeted APC activation in cancer immunotherapy to enhance the abscopal effect. Front Immunol. (2019) 10:604. 10.3389/fimmu.2019.00604 31001249 PMC6454083

[47] Obeid M Tesniere A Ghiringhelli F Fimia GM Apetoh L Perfettini J-L. Calreticulin exposure dictates the immunogenicity of cancer cell death. Nat Med. (2007) 13:54–61. 10.1038/nm1523 17187072

[48] Obeid M Panaretakis T Joza N Tufi R Tesniere A van Endert P. Calreticulin exposure is required for the immunogenicity of γ-irradiation and UVC light-induced apoptosis. Cell Death Differ. (2007) 14:1848–50. 10.1038/sj.cdd.4402201 17657249

[49] Galluzzi L Kepp O Kroemer G. Immunogenic cell death in radiation therapy. Oncoimmunology. (2013) 2:e26536. 10.4161/onci.26536 24404424 PMC3881599

[50] Teitz-Tennenbaum S Li Q Okuyama R Davis MA Sun R Whitfield J. Mechanisms involved in radiation enhancement of intratumoral dendritic cell therapy. J Immunother. (2008) 31:345–58. 10.1097/CJI.0b013e318163628c 18391761 PMC3103774

[51] Tel J Anguille S Waterborg CEJ Smits EL Figdor CG de Vries IJM. Tumoricidal activity of human dendritic cells. Trends Immunol. (2014) 35:38–46. 10.1016/j.it.2013.10.007 24262387 PMC7106406

[52] Srivastava RM Lee SC Andrade Filho PA Lord CA Jie H-B Davidson HC. Cetuximab-Activated natural killer and dendritic cells collaborate to trigger tumor antigen-specific T-cell immunity in head and neck cancer patients. Clin Cancer Res. (2013) 19:1858–72. 10.1158/1078-0432.CCR-12-2426 23444227 PMC3640274

[53] Trivedi S Srivastava RM Concha-Benavente F Ferrone S Garcia-Bates TM Li J. Anti-EGFR targeted monoclonal antibody isotype influences antitumor cellular immunity in head and neck cancer patients. Clin Cancer Res. (2016) 22:5229–37. 10.1158/1078-0432.CCR-15-2971 27217441 PMC5093040

[54] Sneha S Nagare RP Priya SK Sidhanth C Pors K Ganesan TS. Therapeutic antibodies against cancer stem cells: a promising approach. Cancer Immunol Immunother. (2017) 66:1383–98. 10.1007/s00262-017-2049-0 28840297 PMC11028654

[55] Vera M Razquin N Prieto J Melero I Fortes P González-Aseguinolaza G. Intratumoral injection of dendritic cells transduced by an SV40-based vector expressing interleukin-15 induces curative immunity mediated by CD8^+^ T lymphocytes and NK cells. Mol Ther. (2005) 12:950–9. 10.1016/j.ymthe.2005.03.030 15921960

[56] Hu J Yuan X Belladonna ML Ong JM Wachsmann-Hogiu S Farkas DL. Induction of potent antitumor immunity by intratumoral injection of interleukin 23–transduced dendritic cells. Cancer Res. (2006) 66:8887–96. 10.1158/0008-5472.CAN-05-3448 16951206

[57] Kalinski P Mailliard RB Giermasz A Zeh HJ Basse P Bartlett DL. Natural killer–dendritic cell cross-talk in cancer immunotherapy. Expert Opin Biol Ther. (2005) 5:1303–15. 10.1517/14712598.5.10.1303 16197336

[58] Van Elssen CHMJ Oth T Germeraad WTV Bos GMJ Vanderlocht J. Natural killer cells: the secret weapon in dendritic cell vaccination strategies. Clin Cancer Res. (2014) 20:1095–103. 10.1158/1078-0432.CCR-13-2302 24590885

[59] Ferlazzo G Tsang ML Moretta L Melioli G Steinman RM Münz C. Human dendritic cells activate resting natural killer (NK) cells and are recognized via the NKp30 receptor by activated NK cells. J Exp Med. (2002) 195:343–51. 10.1084/jem.20011149 11828009 PMC2193591

[60] Fernandez NC Lozier A Flament C Ricciardi-Castagnoli P Bellet D Suter M. Dendritic cells directly trigger NK cell functions: cross-talk relevant in innate anti-tumor immune responses *in vivo*. Nat Med. (1999) 5:405–11. 10.1038/7403 10202929

[61] Böttcher JP Bonavita E Chakravarty P Blees H Cabeza-Cabrerizo M Sammicheli S. NK cells stimulate recruitment of cDC1 into the tumor microenvironment promoting cancer immune control. Cell. (2018) 172:1022–37.e14. 10.1016/j.cell.2018.01.004 29429633 PMC5847168

[62] Barry KC Hsu J Broz ML Cueto FJ Binnewies M Combes AJ. A natural killer-dendritic cell axis defines checkpoint therapy-responsive tumor microenvironments. Nat Med. (2018) 24:1178–91. 10.1038/s41591-018-0085-8 29942093 PMC6475503

[63] Liu C Lou Y Lizée G Qin H Liu S Rabinovich B. Plasmacytoid dendritic cells induce NK cell-dependent, tumor antigen-specific T cell cross-priming and tumor regression in mice. J Clin Invest. (2008) 118:1165–75. 10.1172/JCI33583 18259609 PMC2230660

[64] Gerosa F Baldani-Guerra B Nisii C Marchesini V Carra G Trinchieri G. Reciprocal activating interaction between natural killer cells and dendritic cells. J Exp Med. (2002) 195:327–33. 10.1084/jem.20010938 11828007 PMC2193595

[65] Martín-Fontecha A Thomsen LL Brett S Gerard C Lipp M Lanzavecchia A Induced recruitment of NK cells to lymph nodes provides IFN-γ for TH1 priming. Nat Immunol. (2004) 5:1260–5. 10.1038/ni1138 15531883

[66] Van Tendeloo VF Van de Velde A Van Driessche A Cools N Anguille S Ladell K. Induction of complete and molecular remissions in acute myeloid leukemia by Wilms' tumor 1 antigen-targeted dendritic cell vaccination. Proc Natl Acad Sci USA. (2010) 107:13824–9. 10.1073/pnas.1008051107 20631300 PMC2922237

[67] Aparicio LMA Fernandez IP Cassinello J. Tyrosine kinase inhibitors reprogramming immunity in renal cell carcinoma: rethinking cancer immunotherapy. Clin Transl Oncol. (2017) 19:1175–82. 10.1007/s12094-017-1657-7 28409322 PMC5599454

[68] Rizell M Sternby Eilard M Andersson M Andersson B Karlsson-Parra A Suenaert P. Phase 1 trial with the cell-based immune primer ilixadencel, alone, and combined with sorafenib, in advanced hepatocellular carcinoma. Front Oncol. (2019) 9:19. 10.3389/fonc.2019.00019 30719425 PMC6348253

[69] Huang C Ramakrishnan R Trkulja M Ren X Gabrilovich DI. Therapeutic effect of intratumoral administration of DCs with conditional expression of combination of different cytokines. Cancer Immunol Immunother. (2012) 61:573–9. 10.1007/s00262-011-1198-9 22223258 PMC4467209

[70] Yang S-C Hillinger S Riedl K Zhang L Zhu L Huang M. Intratumoral administration of dendritic cells overexpressing CCL21 generates systemic antitumor responses and confers tumor immunity. Clin Cancer Res. (2004) 10:2891–901. 10.1158/1078-0432.CCR-03-0380 15102698

[71] Liu S Yu Y Zhang M Wang W Cao X The involvement of TNF-related apoptosis-inducing ligand in the enhanced cytotoxicity of IFN-stimulated human dendritic cells to tumor cells. J Immunol. (2001) 166:5407–15. 10.4049/jimmunol.166.9.5407 11313377

[72] Kalinski P Urban J Narang R Berk E Wieckowski E Muthuswamy R. Dendritic cell-based therapeutic cancer vaccines: what we have and what we need. Future Oncol. (2009) 5:379–90. 10.2217/fon.09.6 19374544 PMC2713774

[73] Möller I Michel K Frech N Burger M Pfeifer D Frommolt P. Dendritic cell maturation with poly(I:C)-based versus PGE2-based cytokine combinations results in differential functional characteristics relevant to clinical application. J Immunother. (2008) 31:506–19. 10.1097/CJI.0b013e318177d9e5 18463533

[74] Koski GK Koldovsky U Xu S Mick R Sharma A Fitzpatrick E. A novel dendritic cell-based immunization approach for the induction of durable Th1-polarized anti-HER-2/neu responses in women with early breast cancer. J Immunother. (2012) 35:54–65. 10.1097/CJI.0b013e318235f512 22130160 PMC3241864

[75] Bonnotte B Crittenden M Larmonier N Gough M Vile RG. MIP-3alpha transfection into a rodent tumor cell line increases intratumoral dendritic cell infiltration but enhances (facilitates) tumor growth and decreases immunogenicity. J Immunol. (2004) 173:4929–35. 10.4049/jimmunol.173.8.4929 15470034

[76] Furumoto K Soares L Engleman EG Merad M. Induction of potent antitumor immunity by *in situ* targeting of intratumoral DCs. J Clin Invest. (2004) 113:774–83. 10.1172/JCI200419762 14991076 PMC351319

[77] Shurin GV Ouellette CE Shurin MR. Regulatory dendritic cells in the tumor immunoenvironment. Cancer Immunol Immunother. (2012) 61:223–30. 10.1007/s00262-011-1138-8 22065047 PMC3314382

[78] Zong J Keskinov AA Shurin G V Shurin MR. Tumor-derived factors modulating dendritic cell function. Cancer Immunol Immunother. (2016) 65:821–33. 10.1007/s00262-016-1820-y 26984847 PMC11028482

[79] Udagawa M Kudo-Saito C Hasegawa G Yano K Yamamoto A Yaguchi M. Enhancement of immunologic tumor regression by intratumoral administration of dendritic cells in combination with cryoablative tumor pretreatment and bacillus calmette-guerin cell wall skeleton stimulation. Clin Cancer Res. (2006) 12:7465–75. 10.1158/1078-0432.CCR-06-1840 17189420

[80] López-Relaño J Martín-Adrados B Real-Arévalo I Lozano-Bartolomé J Abós B Sánchez-Ramón S. Monocyte-derived dendritic cells differentiated in the presence of lenalidomide display a semi-mature phenotype, enhanced phagocytic capacity, and th1 polarization capability. Front Immunol. (2018) 9:1328. 10.3389/fimmu.2018.01328 29951065 PMC6008535

[81] Santini SM Lapenta C Logozzi M Parlato S Spada M Di Pucchio T. Type I interferon as a powerful adjuvant for monocyte-derived dendritic cell development and activity *in vitro* and in Hu-PBL-SCID mice. J Exp Med. (2000) 191:1777–88. 10.1084/jem.191.10.1777 10811870 PMC2193160

[82] Santodonato L D'Agostino G Nisini R Mariotti S Monque DM Spada M. Monocyte-derived dendritic cells generated after a short-term culture with IFN-alpha and granulocyte-macrophage colony-stimulating factor stimulate a potent Epstein-Barr virus-specific CD8+ T cell response. J Immunol. (2003) 170:5195–202. 10.4049/jimmunol.170.10.5195 12734367

[83] Lapenta C Santini SM Logozzi M Spada M Andreotti M Di Pucchio T. Potent immune response against HIV-1 and protection from virus challenge in hu-PBL-SCID mice immunized with inactivated virus-pulsed dendritic cells generated in the presence of IFN-α. J Exp Med. (2003) 198:361–7. 10.1084/jem.20021924 12874266 PMC2194078

[84] Spadaro F Lapenta C Donati S Abalsamo L Barnaba V Belardelli F IFN-a enhances cross-presentation in human dendritic cells by modulating antigen survival, endocytic routing, and processing. Blood. (2012) 119:1407–17. 10.1182/blood-2011-06-363564 22184405

[85] Santini SM Lapenta C Donati S Spadaro F Belardelli F Ferrantini M. Interferon-α-conditioned human monocytes combine a Th1-orienting attitude with the induction of autologous Th17 responses: role of IL-23 and IL-12. PLoS ONE. (2011) 6:e17364. 10.1371/journal.pone.0017364 21387004 PMC3046151

[86] Lapenta C Donati S Spadaro F Castaldo P Belardelli F Cox MC. NK cell activation in the antitumor response induced by IFN-α dendritic cells loaded with apoptotic cells from follicular lymphoma patients. J Immunol. (2016) 197:795–806. 10.4049/jimmunol.1600262 27357153

[87] Havel JJ Chowell D Chan TA. The evolving landscape of biomarkers for checkpoint inhibitor immunotherapy. Nat Rev Cancer. (2019) 19:133–50. 10.1038/s41568-019-0116-x 30755690 PMC6705396

[88] Yarchoan M Hopkins A Jaffee EM. Tumor mutational burden and response rate to PD-1 inhibition. N Engl J Med. (2017) 377:2500–1. 10.1056/NEJMc1713444 29262275 PMC6549688

[89] Topalian SL Taube JM Anders RA Pardoll DM. Mechanism-driven biomarkers to guide immune checkpoint blockade in cancer therapy. Nat Rev Cancer. (2016) 16:275–87. 10.1038/nrc.2016.36 27079802 PMC5381938

[90] Gibney GT Weiner LM Atkins MB. Predictive biomarkers for checkpoint inhibitor-based immunotherapy. Lancet Oncol. (2016) 17:e542–51. 10.1016/S1470-2045(16)30406-5 27924752 PMC5702534

[91] Tumeh PC Harview CL Yearley JH Shintaku IP Taylor EJM Robert L. PD-1 blockade induces responses by inhibiting adaptive immune resistance. Nature. (2014) 515:568–71. 10.1038/nature13954 25428505 PMC4246418

[92] Riaz N Havel JJ Makarov V Desrichard A Urba WJ Sims JS. Tumor and microenvironment evolution during immunotherapy with nivolumab. Cell. (2017) 171:934–49.e16. 10.1016/j.cell.2017.09.028 29033130 PMC5685550

[93] Chowell D Morris LGT Grigg CM Weber JK Samstein RM Makarov V. Patient HLA class I genotype influences cancer response to checkpoint blockade immunotherapy. Science. (2018) 359:582–7. 10.1126/science.aao4572 29217585 PMC6057471

[94] Sivan A Corrales L Hubert N Williams JB Aquino-Michaels K Earley ZM. Commensal Bifidobacterium promotes antitumor immunity and facilitates anti-PD-L1 efficacy. Science. (2015) 350:1084–9. 10.1126/science.aac4255 26541606 PMC4873287

[95] Vétizou M Pitt JM Daillère R Lepage P Waldschmitt N Flament C. Anticancer immunotherapy by CTLA-4 blockade relies on the gut microbiota. Science. (2015) 350:1079–84. 10.1126/science.aad1329 26541610 PMC4721659

[96] Spranger S Dai D Horton B Gajewski TF. Tumor-residing Batf3 dendritic cells are required for effector T cell trafficking and adoptive T cell therapy. Cancer Cell. (2017) 31:711–23.e4. 10.1016/j.ccell.2017.04.003 28486109 PMC5650691

[97] Garris CS Arlauckas SP Kohler RH Trefny MP Garren S Piot C. Successful anti-PD-1 cancer immunotherapy requires T cell-dendritic cell crosstalk involving the cytokines IFN-γ and IL-12. Immunity. (2018) 49:1148–61.e7. 10.1016/j.immuni.2018.09.024 30552023 PMC6301092

[98] Chow MT Ozga AJ Servis RL Frederick DT Lo JA Fisher DE. Intratumoral activity of the CXCR3 chemokine system is required for the efficacy of anti-PD-1 therapy. Immunity. (2019) 50:1498–512.e5. 10.1016/j.immuni.2019.04.010 31097342 PMC6527362

[99] Binnewies M Mujal AM Pollack JL Combes AJ Hardison EA Barry KC. Unleashing type-2 dendritic cells to drive protective antitumor CD4^+^ T cell immunity. Cell. (2019) 177:556–71.e16. 10.1016/j.cell.2019.02.005 30955881 PMC6954108

